# The Role of Quorum Sensing Mechanism in the Functional Properties of Lactic Acid Bacteria

**DOI:** 10.3390/microorganisms14030618

**Published:** 2026-03-10

**Authors:** Annalaura Iodice, Giuseppina Tommonaro

**Affiliations:** Institute of Biomolecular Chemistry (ICB), National Council of Research (CNR), Via Campi Flegrei, 34, 80078 Pozzuoli, NA, Italy; annalauraiodice@cnr.it

**Keywords:** lactic acid bacteria (LAB), quorum sensing (QS), bacteriocin, quorum sensing inhibition (QSI)

## Abstract

Lactic acid bacteria (LAB) are non-spore-forming, non-respiring, Gram-positive cocci or rods that produce lactic acid through carbohydrate fermentation. They are widely used in food and dairy production as probiotics, biofertilizers, and as sources of industrially valuable exopolysaccharides. Growing evidence indicates that many of these functional properties are regulated by quorum sensing (QS), a cell–cell communication mechanism that coordinates bacterial behavior in response to population density. This review summarizes current knowledge on the role of QS in regulating key physiological and functional traits of LAB, including biofilm formation, stress adaptation, metabolite production, and host interactions. Additionally, it highlights the ability of LAB-derived molecules to interfere with QS systems of pathogenic bacteria, contributing to pathogen control. Overall, this review emphasizes QS as a key regulatory mechanism underlying the technological and probiotic potential of LAB, with important implications for food, health, and biotechnological applications.

## 1. Lactic Acid Bacteria (LAB)—An Overview

Lactic acid bacteria (LAB) are a heterogeneous group of Gram-positive, non-spore-forming, acid-tolerant bacteria that are predominantly non-motile, although several motile species have been described [1].

These bacteria, which belong to the phylum Firmicutes, class Bacilli, order Lactobacillales, are widely distributed in environmental niches and are common members of plant, animal, and human microbiota, including both beneficial and pathogenic species [2,3,4]. Historically classified based on phenotypic traits, LAB taxonomy has undergone extensive revision with the advent of molecular approaches, particularly 16S rRNA gene analysis and, more recently, genome-based methods. A comprehensive polyphasic reclassification reorganized more than 460 LAB species into a single family, Lactobacillaceae, comprising 35 genera, many of which were newly established [5,6,7,8,9,10].

Based on their fermentative metabolism and phylogenetic relationships, LAB are classified into two subgroups: homofermentative and heterofermentative. These subgroups use substantially different strategies for energy generation and, due to their distinct substrate preferences, coexist in multiple ecological niches [11].

Beyond primary metabolism, LAB are notable for producing a wide range of bioactive metabolites and enzymes, including vitamins, bacteriocins, gamma-aminobutyric acid (GABA), exopolysaccharides, flavor compounds, and hydrolytic enzymes, which underpin their significant biotechnological value [12,13,14,15,16,17,18,19].

LAB are extensively applied in the food industry as starter cultures for dairy and non-dairy fermentation, where they enhance food safety, shelf life, nutritional quality, and sensory properties. Many food-associated LAB have a long history of safe use and are recognized as generally regarded as safe (GRAS) or qualified presumption of safety (QPS) organisms by the European Food Safety Authority and the US Food and Drug Administration, respectively [20,21]. Their antimicrobial activity is primarily attributed to organic acid production and the synthesis of inhibitory compounds such as bacteriocins, hydrogen peroxide, and diacetyl [22,23,24,25,26,27,28].

Several LAB species also exhibit probiotic properties, contributing to gut health through pathogen inhibition, immune modulation, improvement of lactose digestion, and reduction in cholesterol levels. Additionally, LAB-derived metabolites have demonstrated promising nutraceutical and anticancer potential in vitro and in animal models, although further clinical validation is required [29,30,31,32,33].

Beyond food and health applications, LAB are increasingly explored in sustainable agriculture as biofertilizers, biostimulants, and biocontrol agents. They promote plant growth through phytohormone production, nutrient solubilization, and stress tolerance enhancement, while suppressing phytopathogens via antimicrobial metabolites. Overall, the physiological versatility, metabolic diversity, and functional properties of LAB make them valuable resources across food, health, and agricultural biotechnology [34,35,36,37,38,39,40,41,42,43].

More recently, LAB have also demonstrated capabilities in reducing and degrading toxic contaminants, including heavy metals [44], mycotoxins [45], cyanotoxins [46], and organophosphorus pesticides (OPPs) [47].

A lesser-known but increasingly studied aspect of lactic acid bacteria biology is their ability to produce secondary metabolites that act either as autoinducers—signaling molecules involved in the microbial cell-to-cell communication process known as quorum sensing (QS)—or as quorum sensing inhibitors (QSIs), typically compounds that can interfere with the QS systems of other bacterial species, especially pathogens whose biofilm formation and virulence factor production are regulated by QS mechanisms.

## 2. Quorum Sensing

Quorum sensing (QS) is a microbial intercellular communication mechanism that enables bacteria to synchronously coordinate their behavior in response to fluctuations in cell density and species composition in neighboring communities [48].

Many microbial physiological functions, such as virulence [49], bioluminescence [50], horizontal transfer of DNA [51], synthesis of antibiotics and bacteriocins, ability to resist to the harsh gastric stress [52], production of secondary metabolites [53] and enzymes [54], drug resistance, formation of biofilms [55], and alteration of foodstuffs, are regulated by QS mechanisms [56]. However, this mechanism could be affected by different factors [57,58,59,60].

At the basis of this mechanism there is the production of signaling molecules (QSM), usually named autoinducers (AI), as well as their detection, and the resulting cellular response. As the bacterial population increases, the concentration of autoinducer molecules rises progressively in the extracellular environment. Once a critical concentration threshold is reached, the bacteria can detect autoinducer molecules and initiate a specific response [48].

Based on their chemical nature, autoinducers bind specific membrane or cytosolic receptors, leading, ultimately, to the transcriptional activation or repression of defined target genes.

To be considered as a QSM, autoinducers must: (a) be synthesized in response to specific physiological conditions, (b) accumulate in the extracellular environment and recognize a specific receptor, (c) trigger a cellular response upon reaching a critical threshold concentration, and (d) be degraded or inactivated following the completion of the physiological process [61].

**Figure 1:**
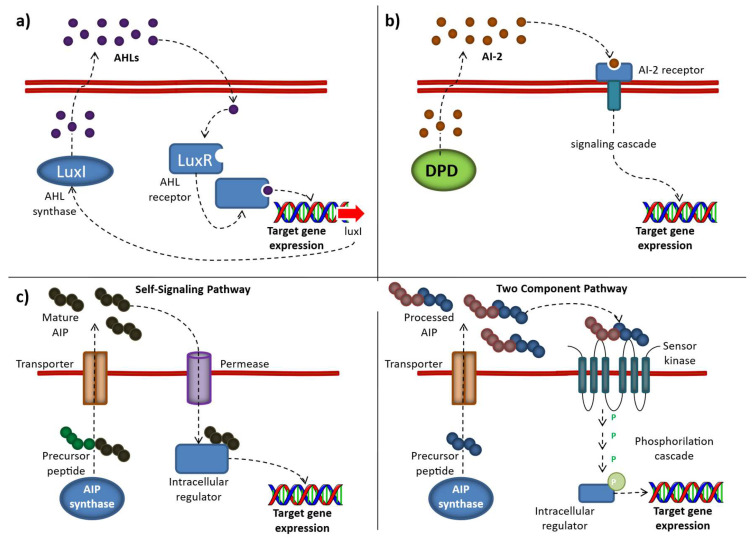
Different QS mechanisms, including the produced autoinducers: (**a**) AI-1. The AI-1 system relies on AHL synthases (LuxI or LuxM), which generate acyl-homoserine lactones (AHLs) from S-adenosylmethionine (SAM). These signaling molecules are secreted into the extracellular environment and, once they accumulate to a threshold concentration, re-enter the cell and bind to LuxR-type intracellular receptors. (**b**) AI-2. Autoinducer-2 (AI-2) is produced as a byproduct of the activated methyl cycle. Its precursor, 4,5-dihydroxy-2,3-pentanedione (DPD), can spontaneously rearrange into either the R- or S- forms of 2-methyl-2,3,3,4-tetrahydroxytetrahydrofuran which can form borate complexes in the presence of environmental boron. (**c**) AIP (“Self-Signaling” and “Two Component” pathways) mechanisms. Autoinducing peptides (AIPs) are released into the extracellular environment, where they accumulate until reaching a threshold concentration. In the “self-signaling pathway,” they are taken back into the cell through an oligopeptide transporter system. In contrast, in the “two-component pathway,” AIPs bind to and activate a membrane-bound histidine kinase receptor, which subsequently transfers a phosphate group to an intracellular response regulator, leading to enhanced expression of the target gene. (Source: [66] Abbamondi, G.R.; Tommonaro, G. Research Progress and Hopeful Strategies of Application of Quorum Sensing in Food, Agriculture and Nanomedicine. Microorganisms 2022, 10, 1192. https://doi.org/10.3390/microorganisms10061192).

The main classes of autoinducers involved in this bacterial phenomenon are: acyl-homoserine lactones (AHLs), autoinducer peptides (AIP), and autoinducer-2 (AI-2) (Figure 1).

### 2.1. Acyl-Homoserine Lactones

Acyl-homoserine lactones (AHLs), also known as autoinducers 1 (AI-1) and produced by Gram-negative bacteria, are the most investigated signal molecules. Their chemical structure consists of a homoserine lactone ring (HSL) and hydrophobic acyl side chain. AHL-mediated QS systems possess multiple signal-receptor homologues including LuxI/R, LasI/R, RhlI/R, AfeI/R, BtaI/R, EsaI/R, and TofI/R [62].

### 2.2. Autoinducer Peptides

The autoinducer peptides (AIP) are primarily produced by Gram-postive bacteria, including LAB. Autoinducer peptides (AIP), characterized by a large structural diversity, are synthesized by ribosomes as precursor peptides and, to become active and stable, they undergo post-translational modifications during excretion, a process typically facilitated by a membrane-associated ATP-binding cassette (ABC) transporter. As the population density increases, the AIPs accumulate in the environment and, when threshold level is reached, bind their specific membrane-associated histidine kinase receptor, leading to its activation via autophosphorylation on a conserved histidine residue. Subsequently, the activated receptor kinase transfers the phosphoryl group to a conserved aspartate residue of the intracellular response regulator, which, in turn, becomes activated and thereby influences the transcription of target genes, including the AIP genes, genes for the receptor kinase, and response regulator and genes for the ABC transporter [63].

### 2.3. Autoinducer-2

The family of autoinducers, widespread in the bacterial world—both in Gram-positive and Gram-negative species and involved in interspecies communication—is generically referred to as autoinducer-2 (AI-2). Autoinducer-2 (AI-2) molecules are chemically characterized as furanosyl borate diesters produced by LuxS enzyme from a common precursor, 4,5-dihydroxy-2,3 pentanedione (DPD). AI-2 is produced from S-adenosylmethionine (SAM) in three enzymatic steps. The use of SAM as a methyl donor in these and other metabolic processes produces the toxic intermediate S-adenosylhomocysteine (SAH), which is hydrolyzed to S-ribosylhomocysteine (SRH) and adenine by the nucleosidase enzyme Pfs (5′methylthioadenosine/S-adenosylhomocysteine nucleosidase). LuxS catalyzes the cleavage of SRH to 4,5-dihydroxy 2,3-pentanedione (DPD) and homocysteine [64]. DPD undergoes spontaneous rearrangements to produce a collection of interconverting molecules, which allow bacteria to respond to endogenously produced AI-2, and also to AI-2 produced by other bacterial species in the vicinity, giving rise to the idea that AI-2 represents a universal language: a “Bacterial Esperanto” [65] (Figure 1).

## 3. Quorum Sensing in LAB

The main autoinducers detected in LAB are AIPs—products of oligopeptide cleavage/modification that regulate gene transcription via a two-component system (TCS) as well as AI-2.

Studies conducted over the past 20 years have shown that QS regulates a variety of metabolic and physiological activities in lactic acid bacteria such as biofilm formation [67], autolysis, bacteriocin and exopolysaccharides (EPS) biosynthesis, as well as stress tolerance [68]. Moreover, it is shown that QS has a crucial role in the regulation of the probiotic function of LAB strains [69].

For instance, Buck et al. [70] demonstrated that *Lactobacillus acidophilus* during planktonic growth produces the autoinducer AI-2, which, through a QS-related mechanism, allows the bacterium to adapt to the harsh conditions of the human digestive tract. Specifically, AI-2 affects the ability of *Lb. acidophilus* to adhere to the intestinal epithelium, promoting both retention in the gastrointestinal tract and interactions with the host immune system. The study suggests that the LuxS-deficient strain of *Lb. acidophilus* did not produce AI-2, resulting in a significant loss of adhesive ability to Caco-2 epithelial cells when the population was harvested during the logarithmic phase of growth [70].

Instead, other studies show the implications of the QS system in the biosynthesis of bacteriocins by lactic acid bacteria.

### 3.1. Involvement of QS in the Synthesis of Bacteriocins

LAB’s bacteriocins are antimicrobial peptides—with different bactericidal and bacteriostatic activity—synthesized during the logarithmic growth phase. Their mechanism of action is based on disrupting cell membrane integrity and/or inhibiting protein and nucleic acid synthesis, making the development of resistance challenging [71]. Most of them are used against phylogenetically related bacteria and against some pathogens such as *Bacillus*, *Clostridium*, and *Listeria*, while others have shown antiviral, antifungal, and antiprotozoal (antileishmanial) activities [72,73].

Based on molecular weight, chemical structure, thermal stability, and post-translational modifications, these bioactive molecules are divided into two major groups: (1) Class I, which includes small bacteriocins (less than 10 kDa) that undergo posttranslational modifications after ribosomal synthesis. The most extensively studied bacteriocins within Class I are Lantibiotics (Lanthipeptides), small peptides typically characterized by the residues of special amino acids like lanthionine, heterocyclic structures, head-to-tail cyclization, and glycosylation. (2) Class II includes bacteriocins (greater than 10 kDa) non-modified after ribosomal synthesis, heat-stable, and non-thiol peptides. Most of them are cationic peptides, and their antimicrobial activity is primarily exerted by enhancing membrane permeability. They are further classified into four subclasses based on their structural features [74]. Their synthesis is often coordinated by a QS mechanism, typically an AIPs-two component system (AIPs-TCS) composed of two entities: histidine kinase (LanK) and a transcriptional regulator (LanR), or the autoinducer is the bacteriocin itself (Class I). Instead, the production of Class II bacteriocins is regulated by a three-component system, where the signaling molecule is a peptide pheromone rather than the bacteriocin, unlike in lantibiotics [75].

In LAB, sometimes, bacteriocin I/lantibiotic itself acts as the autoinducer [76].

A well-known example is the QS system involved in the synthesis of the antibacterial peptide nisin (Class I bacteriocin) in *Lactococcus lactis*, in which nisin itself acts as an AIP signaling molecule to induce the expression of the entire nis cluster, which contains more than 10 genes [77]. These genes are involved in response monitoring, protein modification, transmembrane signal transduction, self-immunity, ABC transporter synthesis, and synthesis of extracellular proteases for the removal of leader peptides. In contrast, for Class II bacteriocins, unlike lantibiotics, the signaling stimulus is a peptide pheromone other than bacteriocins [61].

**Figure 2:**
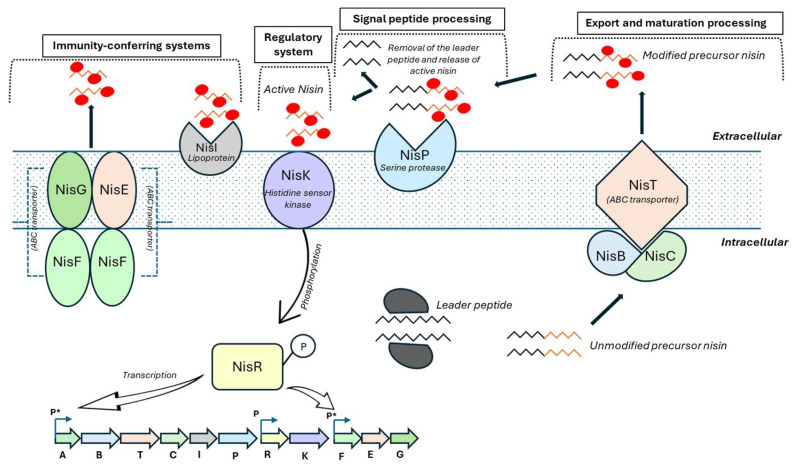
Biosynthesis, regulation, and immunity of nisin in *Lactococcus lactis*. Nisin is synthesized as a ribosomal precursor peptide (NisA) composed of an N-terminal leader and a C-terminal core peptide. The precursor is modified by the dehydratase NisB and the cyclase NisC, which catalyzes dehydration of serine and threonine residues and subsequent formation of lanthionine rings, respectively. The fully modified precursor is exported by the ABC transporter NisT and extracellularly processed by the serine protease NisP to release active nisin. Immunity of the producing strain is mediated by the lipoprotein NisI and the ABC transporter complex NisFEG. Extracellular nisin activates the two-component regulatory system NisK/NisR, leading to phosphorylation of NisR and induction of transcription from nisin-responsive promoters (P*), while other promoters (P) are constitutively active. (Adapted from [78]. Chen J, van Heel AJ, Kuipers OP. 2020. Subcellular localization and assembly process of the nisin biosynthesis machinery in *Lactococcus lactis*. mBio 11:e02825-20. https://doi.org/10.1128/mBio.02825-20).

The regulatory system controlling nisin production has been extensively characterized in *Lb*. *lactis*. Expression of the nisin operon (nisABTCIPRK) is regulated in a cell density-dependent manner and responds to extracellular nisin concentrations. The two-component regulatory system consists of the histidine kinase NisK and the response regulator NisR. At low nisin concentrations, NisR remains inactive in its dephosphorylated state. NisA, the nisin precursor peptide, undergoes dehydration by NisB and cyclization by NisC. The protease NisP cleaves the N-terminal leader peptide to generate mature nisin, which is then exported from the cell by the ABC transporter NisT. When the extracellular nisin reaches a threshold concentration, it activates the intracellular domain of NisK, leading to the phosphorylation of NisR. This phosphorylation cascade induces transcription of the genes required for nisin biosynthesis, as well as immunity genes that protect the producing strain from its own bacteriocin, including *nisI* and the ABC transporter genes *nisGEF* (Figure 2).

Moreover, it was discovered that the lactic acid bacterium *Carnobacterium piscicola* LV17 possesses a CS (autoinducer)-CbnK (histidine protein kinase)-CbnR (response regulator) three-component QS system involved in the biosynthesis of three carnobacteriocins (bacteriocins) with potent antimicrobial activity against several Gram-positive bacteria, including important human pathogens and bacteria with high food spoilage properties [79].

Similar types of QS mechanisms involved in bacteriocins production have also been described in *Lactiplantibacillus plantarum* and *Lb. salivarius*, which produce several plantaricins, as well as in *Latilactobacillus sakei* LTH673, which produces the lacocin P [61].

In contrast, it has been discovered that bacteriocin synthesis in *Lactobacillus plantarum NMD-17* is regulated by a LuxS/AI-2-mediated QS system [80].

### 3.2. Involvement of QS in: Biofilm Formation, Stress Tolerance, and Exopolysaccharide Synthesis

In lactic acid bacteria, AI-2 signaling serves as a mediator of interactions among several species in dual-species cultures, thereby promoting enhanced biofilm formation and improving resistance to unfavorable environmental factors, including pH fluctuations, temperature extremes, and antimicrobial agents [81].

The LuxS/AI-2 QS system also regulates the expression of genes responsible for exopolysaccharides (EPS) production, and for other macromolecules essential for biofilm stability and structural integrity. These macromolecules, in fact, are essential in maintaining surface adhesion, cell cohesion, and the three-dimensional structure of the biofilm, while also providing protection against extrinsic factors [81,82].

For instance, it was shown that deletion of the *luxS* gene in *Lacticaseibacillus rhamnosus* GG significantly impairs biofilm formation, but adding synthetic DPD (the AI-2 precursor) or supernatant from the wild-type strain can reverse this defect [55].

In addition, AI-2 signaling enhances bile salt tolerance in *Lb. plantarum*, *Fructilactobacillus sanfranciscensis*, and *Lb. paraplantarum* by promoting biofilm formation and EPS production, thereby enabling survival in physiological bile concentrations and intestinal fluids [67,83,84].

In *Lcb. rhamnosus* GG, a human isolate producing AI-2-like molecules, the *luxS* gene appears to be located within an operon together with yxjH, which encodes a putative cobalamin-independent methionine synthase. In silico analysis identified a methionine-specific T-box element in the leader region of the proposed yxjH–luxS operon. However, transcriptional analyses demonstrated that *luxS* is predominantly expressed as a monocistronic transcript. Functional characterization using a *luxS* knockout mutant confirmed that *luxS* is responsible for AI-2 production in *Lcb. rhamnosus* GG. Deletion of *luxS* also caused pleiotropic effects on the growth of this strain. Partial restoration of growth was achieved by supplementation with cysteine, pantothenate, folic acid, and biotin, indicating a central role of *luxS* in cellular metabolism. Notably, the *luxS* mutant additionally exhibited impaired monospecies biofilm formation [85].

Although, it is now well established that biofilm development depends on AI-2 signaling, the precise signal transduction processes differ among species of LAB, depending on strain-specific features and environmental factors. Consequently, further studies will be required to completely elucidate these dynamics across LAB species [81].

### 3.3. QS in Fermented Food

It was observed that, in *Lactiplantibacillus plantarum* L3, a LuxS/AI-2 Qs mechanism is linked to phenyl lactic acid production, a bioactive compound that inhibits fungal growth in fermented foods [86].

Finally, it was discovered that in *Lb. delbrueckii* subsp. *bulgaricus* a QS system, based on AIP-TCS (LBUL_RS00115/LBUL_RS00110), is involved in autolysis, a common phenomenon in which spontaneous cellular lysis occurs to maintain a favorable environment and prevent excessive cell density in the fermentation medium. This process can improve the quality of fermented dairy products as the intracellular enzymes (proteases, lipases), released in the fermentation medium after cell lysis decompose the inherent proteins and fats in milk, shaping the texture, flavor, physical and chemical properties, and shelf life of fermented dairy products [87].

The quality of fermented foods largely depends on microbial metabolic processes, which are tightly regulated by QS mechanisms. The quality of fermented vegetable-based foods is affected by the composition and microbial succession. Park et al., 2016 [88] evaluated AI-2 activity in the lactic fermented food kimchi and differences in AI-2 signaling intensity were observed. To identify the source of this signal, they isolated 229 lactic acid bacteria from kimchi samples and assessed the AI-2 characteristics of each isolate.

Findings revealed that dominant species within the genera *Lactobacillus*, *Weissella*, and *Leuconostoc* either produced or inhibited AI-2 signals. None of the isolates belonging to the dominant species *Latilactobacillus sakei* (75 isolates) and *Latilactobacillus curvatus* (28 isolates) exhibited AI-2 production, whereas AI-2 inhibition was not observed in any of the 31 *Lpb*. *plantarum* isolates. Overall, these results indicate that the AI-2 activity detected in kimchi likely arises from interactions among the associated microbial food cultures during fermentation and suggested that such foods may act as sources of AI-2 signaling molecules through their characteristic microbial communities [88]. Sourdoughs used for bread leavening consist of diverse communities of lactic acid bacteria (LAB) and yeasts. The composition of the sourdough microbiota, together with the enzymatic activity of the cereal components, plays a key role in determining bread quality [89]. A better understanding of QS in sourdough ecosystems could provide valuable insights into microbial succession during fermentation and its relationship with final bread quality. The AI-2 activity and autoinducing peptide (AIP) production of *Lpb*. *plantarum* isolates during co-cultivation with other LAB have been examined. Co-cultivation with *Fructilactobacillus sanfranciscensis* and *Furfurilactobacillus rossiae* stimulated both AI-2 activity and *luxS* expression in *Lpb*. *plantarum*. Moreover, when grown alongside various sourdough-associated LAB, *Lpb*. *plantarum* showed enhanced production of the AIP plantaricin [90,91]. The recent study by Zhai et al. [92] investigated the impact of exogenous autoinducer-2 (AI-2) activity on the functional properties of *Limosilactobacillus fermentum* 332 and the quality of fermented sausages. The study demonstrated that supplementing AI-2 (up to 100 μmol/L) improved the antioxidant capacity of *Lim*. *fermentum* 332 by enhancing hydroxyl radical scavenging and inhibiting lipid peroxidation, whereas reduced AI-2 activity increased nitrite degradation. Higher AI-2 levels also improved sausage texture, lowered water activity, and increased lightness (L* values). In contrast, decreased AI-2 activity favored the proliferation of *Limosilactobacillus* and *Lactiplantibacillus*, stimulating carbohydrate, lipid, and amino acid metabolism and promoting the formation of key volatile flavor compounds such as ethyl hexanoate, linalyl acetate, and ethyl oleate. Overall, these results highlight the central role of AI-2 in modulating LAB metabolism and shaping the quality and sensory attributes of fermented sausages [92].

Current understanding of QS in food fermentation is still limited and largely based on empirical observations. Nevertheless, research clearly shows that microbial communication significantly influences the quality of fermented foods and beverages. QS plays a key role in microbial succession during fermentation, promoting the growth of certain species or strains while inhibiting others, including closely related ones. For example, QS-regulated bacteriocins can be used in cheese production to suppress non-starter lactic acid bacteria responsible for quality defects [93]. QS also governs biofilm formation in many spoilage and pathogenic microorganisms [94].

To optimize fermented food quality, it is essential to understand how QS works—covering the production and uptake of signaling molecules, genetic regulation, species interactions, and the stability and solubility of these molecules within the food matrix. Additionally, quorum quenching—disrupting specific steps in the QS process—offers promising applications. Although mainly studied in the context of food safety, particularly for controlling biofilms, it may also serve as an alternative strategy for food preservation.

## 4. The Role of AI Signal Interference by LAB in the Inhibition of Pathogenic Bacteria

For centuries, lactic acid bacteria have been used in food fermentation, both to improve sensory and nutritional profiles, and to preserve products against spoilage and pathogenic bacteria.

The authors of [95] reported the antibacterial activity of cell-free supernatant (CFS) of *Lacticaseibacillus paracasei* against *S*. *aureus* (33.2 ± 0.6 mm) and *E*. *coli* (29 ± 0.4 mm), evaluated by means of well diffusion method [95]. *Lactobacilli* provides protective or therapeutic benefits by generating antimicrobial substances, including small peptides, bacteriocins, and organic acids such as butyric, acetic, and lactic acids [96].

Nineteen isolates of LAB, including *Lpb*. *plantarum*, *Lpb*. *pentosus*, *Lb*. *acidophilus*, *Lev*. *brevis*, *Pediococcus pentosaceus*, and *Pediococcus acidilactici,* from the bowels of saltwater fish were assessed for their antimicrobial activity against potentially pathogenic Gram-positive and Gram-negative indicator bacteria (*Vibrio harveyi*, *Vibrio cholera*, *Streptococcus iniae*, *Vibrio alginolyticus*, *Vibrio fluvialis*, *Vibrio parahaemolyticus*, *Streptococcus agalactia,* and *Clostridium cochlearium*) showing the ability to inhibit the growth of indicator bacteria, supporting their potential use as biocontrol agents in fisheries [97]. Some members of the genus *Lactobacillus* have been reported to suppress fungal growth. Two LAB samples (ABRIIFBI-6 and ABRIIFBI-7), isolated from fresh traditional fermented yogurt, were able to inhibit the growth of *Aspergillus flavus*. The potential probiotic ABRIIFBI-6 was able to inhibit the growth of *A*. *niger* similarly to *A*. *flavus*, but ABRIIFBI-7 only slightly inhibits the growth of *A*. *niger* [98]. This antifungal activity is attributed to the production of compounds such as phenyllactic acid, p-hydroxyphenyllactic acid, and other antifungal cyclic dipeptides [99]. The production of these compounds, both in vitro and in vivo, can inhibit the growth of pathogenic microbial strains.

Since the pathogenicity of many foodborne bacteria—like *Salmonella enterica*, *Campylobacter jejuni*, *Listeria monocytogenes*, and *Staphylococcus aureus*—is linked to the production of virulence factors (which mediate adhesion to and invasion of host cells) as well as with the formation of biofilms that are both regulated by the quorum sensing system, the use of strategies aimed at inhibiting this communication mechanism represents a promising approach to reducing bacterial pathogenicity and biofilm development without affecting planktonic cell growth, reducing the risk of resistance development this way [100,101]. This approach could represent a promising alternative to conventional antimicrobial strategies in both industrial and clinical settings [102].

Because of general acceptability and safety, LAB, as well as their adaptation to both intestinal and food ecosystems, the ability of foodborne LAB-derived metabolites to modulate the QS systems of other bacteria, specially pathogens, may represent a promising and eco-friendly approach for controlling foodborne pathogens and undesired biofilms without relying on traditional methods that contribute to antimicrobial resistance and the production of toxic by-products.

**Figure 3:**
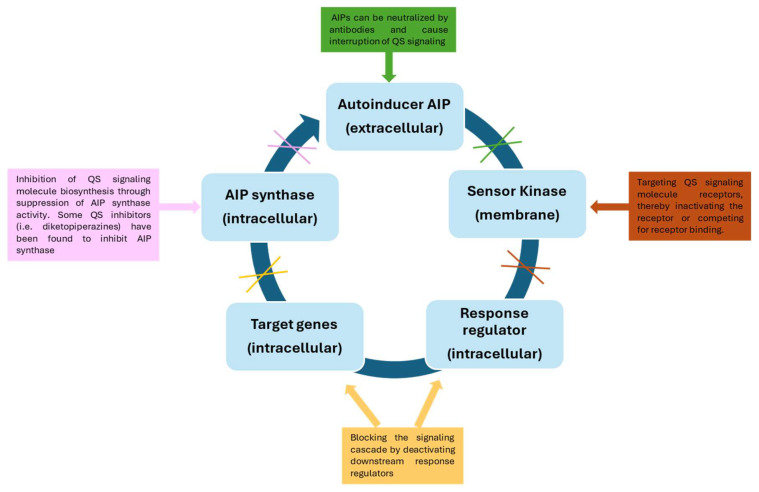
Different strategies for the inhibition of the quorum sensing mechanism.

There are several strategies that can be used to inhibit QS mechanisms, such as: (i) suppression of autoinducer synthesis by using molecules that interfere with the activity of the enzymes responsible for their production or that inhibit the expression of the genes encoding these enzymes; (ii) destruction of signaling molecules once they are released into the extracellular environment, through the use of quorum quenching (QQ) enzymes; (iii) blockage of the signaling molecules’ receptors [103] (Figure 3).

All these approaches could potentially increase bacterial susceptibility to antimicrobial agents, including antibiotics, bacteriocins, and bacteriophages, while simultaneously lowering the risk of resistance development.

Recent studies have demonstrated that several LAB species produce metabolites with quorum sensing inhibition activity (QSI). These metabolites not only inhibit biofilm development but also suppress other virulent traits of pathogenic bacteria.

For example, Park et al. [104] demonstrated in 2014 that a probiotic LAB, *Lat. sakei strain NR28*, isolated from kimchi, has an inhibitory effect on the pathogenicity of enterohaemorrhagic *E. coli* O157:H7 (EHEC) by AI-2 signal interference, without affecting viability. Enterohemorrhagic *E. coli* O157:H7 (EHEC) is a highly contagious foodborne pathogen due to specific dangerous virulence factors, the production of which also involves a LuxS/AI-2 QS system [105]. EHEC’s adhesion to epithelial cells and subsequent colonization may result in hemorrhagic colitis and hemolytic uremic syndrome (HUS) in the human intestine. Moreover, the Shiga toxin (endotoxin) by the same bacterium limits the effectiveness of antibiotic treatment. In this study, we examined the AI-2 associated reducing effect of the putative probiotic strain *Lat*. *sakei* NR28 on the pathogenicity of EHEC, with the purpose of developing a candidate quorum quenching probiotic strain that may support antibiotic therapy and serve to limit the spread of infections. The results suggest that molecules secreted by *Lat. sakei* reduces AI-2 production, motility, biofilm formation, and virulence gene (Type III secretion system) expression in *E. coli* O157:H7 without affecting bacterial viability [104].

Similarly, Pelyuntha et al. (2019) [106] demonstrated that metabolic extracts of *Weissella spp*. from fermented grapes significantly reduced *Salmonella serovars* biofilm formation and AI-2 activity, showing a potential for roles as biocontrol agents to improve microbiological safety in the production of animal source foods (ASF). The mentioned study evaluated the antagonistic properties of 146 LAB strains isolated from 110 fermented food samples (fermented fish, meat, soybean, vegetables, and fruits) against *Salmonella Typhi* and *Salmonella Typhimurium*, which are the causative pathogens of salmonellosis, a severe human infection linked to the consumption of contaminated foods and water and characterized by symptoms such as diarrhea, fever, abdominal cramp, nausea, and vomiting [107]. *Salmonella spp.* utilizes the AI-2/LuxS system to regulate the expression of virulence genes within SPI-1 [108], which is responsible for *Salmonella* host cell invasion. The bacterium enters host cells and involves the intracellular rearrangement of host actin cytoskeleton, leading to food poisoning. The results of the study suggest that metabolites in the CFCS of only two LAB strains (WM33 and WM36) isolated from fermented grapes possess the ability to antagonize and interfere with the growth, biofilm formation, and QS regulation (via AI-2 signaling interference) of *Salmonella* pathogenic indicators. The selected isolates, based on morphological, physiological, and biochemical characteristics, and also on the sequencing analysis of 16s rRNA genes, were identified as *Weissella viridescens* (WM33) and *Weissella confuse* (WM36), previously known as *Lb. viridescens* and *Lb. confusus*, respectively. The chemical investigation indicated organic acids (lactic acid and acetic acid), including 2,4 DTBP (2,4-di-tert-butylphenol), as the main metabolites to act as the anti-salmonella activity of *W*. *confusa* WM36 [106]. They have great potential to be used as biocontrol agents/biopreservatives for controlling *Salmonella* in ASF production to achieve microbiological safety of foods.

More recently, Dimitra Kostoglou et al. [109] had investigated the capacity of foodborne-LAB-derived cell-free supernatants (CFSs) to interfere with AI-2-mediated QS and inhibit biofilm formation in monocultures of two pathogenic bacteria—*Listeria monocytogenes* and *Staphylococcus aureus*.

These pathogenetic bacteria thrive in unclean environments, quickly adapting and multiplying in foods under improper processing and storage, posing serious health risks to consumers [110]. In both pathogens, the AI-2 signaling system is implicated in modulating biofilm formation and virulence; but, they also possess additional QS systems, such as the Agr QS system, which employs auto-inducing peptides (AIPs) to regulate biofilm development and virulence. The study demonstrates that for *L. monocytogenes,* the highest biofilm inhibition is observed when it was allowed to form biofilm in the presence of metabolites produced by the LAB isolates LFMH_B79b and 477 LFMH_B10, which correspond to *Enterococcus faecium* and *Pediococcus acidilactici*, respectively. Regarding *S*. *aureus*, only the CFS from the *E*. *faecium* LFMH_B79b isolate significantly reduced the pathogen’s biofilm formation. Moreover, it should be emphasized that none of the selected CFSs inhibited visible growth of the pathogenetic bacteria under study and this result has supported the hypothesis that the CFSs’ antibiofilm effects are not due to growth suppression but rather reflect a biofilm-specific mechanism—possibly through interference with QS, inhibition of attachment, or disruption of aggregation. It is possible to hypothesize the presence of bioactive cyclic peptides (diketopiperazines) in the CFS. Indeed, the study by Diaz et al. [111] evaluated the influence of lactic acid bacteria supernatant extracts on the growth, biofilm biomass formation, biofilm metabolic activity, and biofilm integrity of the *S. aureus* strains. The chemical analysis of the extracts identified eight 2,5-diketopiperazines, and an in silico docking analysis revealed promising interactions between 2,5-diketopiperazines and key proteins (SarA and AgrA) in *S*. *aureus*, confirming their antivirulence and antibiofilm activities [111]. However, these findings do not exclude the possibility that other regulatory or physicochemical factors unrelated to AI-2 signaling also contribute to the observed antibiofilm effects.

## 5. Conclusions

Current knowledge on quorum sensing communication circuits in lactic acid bacteria has highlighted the critical role of this mechanism in the physiology of these bacteria and consequently, in their related biotechnological applications. However, QS is a highly intricate phenomenon, and the existing literature continue to raise unresolved questions. To date, the most detailed studies in LAB have focused primarily on the regulation of bacteriocin biosynthesis. Ongoing scientific advances are now revealing that QS also governs additional biological functions beyond bacteriocin production. Integrating genomic, metabolomic, and proteomic approaches is expected to significantly expand current knowledge of QS and facilitate its exploitation in applied fields, particularly in the food industry.

## Data Availability

No new data were created or analyzed in this study. Data sharing is not applicable to this article.
